# Effects of High Temperature on Embryological Development and Hormone Profile in Flowers and Leaves of Common Buckwheat (*Fagopyrum esculentum* Moench)

**DOI:** 10.3390/ijms20071705

**Published:** 2019-04-05

**Authors:** Agnieszka Płażek, Aneta Słomka, Przemysław Kopeć, Michał Dziurka, Marta Hornyák, Klaudia Sychta, Jakub Pastuszak, Franciszek Dubert

**Affiliations:** 1 Department of Plant Physiology, University of Agriculture, Podłużna 3, 30-239 Kraków, Poland; rrplazek@cyf-kr.edu.pl (A.P.); marta.golebiewska@gmail.com (M.H.); kubapaaa@gmail.com (J.P.); 2 Department of Plant Cytology and Embryology, Jagiellonian University, Gronostajowa 9, 30-387 Kraków, Poland; aneta.slomka@uj.edu.pl (A.S.); klaudia.michno@doctoral.uj.edu.pl (K.S.); 3 Polish Academy of Sciences, Institute of Plant Physiology, Niezapominajek 21, 30-239 Kraków, Poland; przemyslawkopec@gmail.com (P.K.); dubert@ifr-pan.edu.pl (F.D.)

**Keywords:** abscisic acid, auxin, common buckwheat, cytokinins, embryo sacs, gibberellins, jasmonic acid, pollen grains, thermal stress, salicylic acid

## Abstract

Common buckwheat is a valuable crop, mainly due to the beneficial chemical composition of its seeds. However, buckwheat cultivation is limited because of unstable seed yield. The most important reasons for the low yield include embryo and flower abortion. The aim of this work is to verify whether high temperature affects embryological development in this plant species. The experiment was conducted on plants of a Polish cultivar ‘Panda’ and strain PA15, in which the percentage of degenerating embryo sacs was previously determined and amounted to 32% and 10%, respectively. The plants were cultivated in phytotronic conditions at 20 °C (control), and 30 °C (thermal stress). The embryological processes and hormonal profiles in flowers at various developmental stages (buds, open flowers, and wilted flowers) and in donor leaves were analyzed in two-month-old plants. Significant effects of thermal stress on the defective development of female gametophytes and hormone content in flowers and leaves were observed. Ovules were much more sensitive to high temperature than pollen grains in both genotypes. Pollen viability remained unaffected at 30 °C in both genotypes. The effect of temperature on female gametophyte development was visible in cv. Panda but not in PA15 buds. A drastic reduction in the number of properly developed embryo sacs was clear in open flowers at 30 °C in both genotypes. A considerable increase in abscisic acid in open flowers ready for fertilization may serve as a signal inducing flower senescence observed in the next few days. Based on embryological analyses and hormone profiles in flowers, we conclude that cv. ‘Panda’ is more sensitive to thermal stress than strain PA15, mainly due to a much earlier response to thermal stress involving impairment of embryological processes already in the flower buds.

## 1. Introduction

Common buckwheat (*Fagopyrum esculentum* Moench) belongs to the *Polygonaceae* family, but it is classified as a “pseudocereal” crop due to the cereal-like chemical composition of its seeds. The seeds are gluten-free; have high dietary fiber, high content of rutin and protein, and a well-balanced amino acid composition; and are especially rich in lysine [1,2]. Buckwheat is also used as a fodder crop. The species shows numerous desired properties: it protects soil from erosion, exerts some health effects, efficiently absorbs nitrogen and phosphorus from the soil, is resistant to pests and diseases, and tolerates variable soil conditions. Buckwheat is pollinated primarily by bees and is a source of prized nectar. Common buckwheat seeds are produced mainly in Russia, China, Ukraine, Canada, Poland, Kazakhstan, and the United States of America (USA) (FAOSTAT, 2016). However, the crop has been in a decline for a long time in all countries as a result of low seed production and unstable yield [3]. The cultivation area of buckwheat is limited not only due to low seed yield, as compared with cereals, but also because of the heterogeneity of its maturation, which makes the harvest time difficult to determine [2]. The biggest drawback is the short life of its single flowers, which are only open for one day [4]. Buckwheat is also sensitive to ground frost, high temperatures, and drought, which may cause extensive flower and embryo abortion. It forms dimorphic plants with flowers harboring pistils and stamens of different lengths (pin and thrum types) and shows self-incompatibility [4,5]. Fertilization requires cross-pollination [6,7]. The seed set is insufficient, and it amounts to 15%–53%, depending on the genotype and growth conditions [4,8]. The most important reasons for the low yield include self-incompatibility, insufficient fertilization, embryo abortion, sensitivity to heat, and drought stress, as well as deficiency of assimilates in aging plants [6,9,10]. Slawinska and Obendorf [9] showed that the cultivation of buckwheat plants at a temperature below 25 °C can increase seed formation by up to 40%, while Kreft [11] reported that temperature above 30 °C is deleterious to buckwheat pollen and flowers. The optimal temperature for buckwheat growth is 18–23 °C [4]. Taylor and Obendorf [6] stated that aging plants are less capable of fertilization. The pollen germinates, and the pollen tube is visible, but there is no fusion of gametes. At 35 °C, Guan and Adachi [12] observed ultrastructural changes in embryo sacs of common buckwheat, e.g., accumulation of osmophilic deposits in the egg cell and synergids, collapsed synergids, incomplete membrane systems, and enlarged ribosome/endoplasmic reticulum in the egg cell. Our earlier studies showed that the key problem of the low seed yield in buckwheat is defective development of female gametophytes [8]. The formation of female gametophytes (megasoporogenesis and megagametophytogenesis) was shifted in time as compared with pollen development and occurred in older flowers and at higher temperature. Pollen viability was high, and it effectively germinated on the stigma. Long pollen tubes reaching the ovules were present in buckwheat flowers; thus, pollen was not the reason for the seed formation issues [8]. In other plant species, heat-related elimination of stigma receptivity, inhibition of pollen germination, and pollen tube growth are well known symptoms of temperature stress [13,14,15].

Another problem in buckwheat cultivation is the high degree of abortion of unfertilized flowers. Buckwheat blooming takes a long time and lasts from 30 to 60 days. One plant develops from 500 to 2000 flowers, but only some of them develop into seeds [2,9]. A positive correlation was noticed between the number of flowers and their abortion in all studied cultivars and strains, confirming that the more flowers the plant produced, the more were aborted [8]. 

The course of embryogenesis and flower abortion is under genetic control, but plant hormones and growth regulators, and particularly their concentrations, also play an important role. Auxins, gibberellins, cytokinins, polyamines, abscisic acid, salicylic acid, and jasmonic acid determine the growth and condition of stamens and pistils [16]. Bernier et al. [16] argued that flowering is affected by both plant hormones and sugars—mainly sucrose levels in the leaves that supply nutrients to the apical meristems. 

The aim of this work is to investigate the hormonal profile during flower development associated with the embryological development of common buckwheat exposed to thermal stress (30 °C). A Polish cultivar ‘Panda’ and strain PA15 were chosen based on our earlier experiments [8]. The cultivar, ‘Panda’, showed 32% degenerated embryo sacs, while in strain PA15, disturbances occurred in 10% of flowers. We compared the changes in hormonal profiles at three developmental stages of flower—buds, open flowers, and wilted flowers—with the embryological development of pollen grains and embryo sacs.

## 2. Results

### 2.1. Embryological Analyses

#### 2.1.1. Pollen Viability and Development

**Figure 1:**
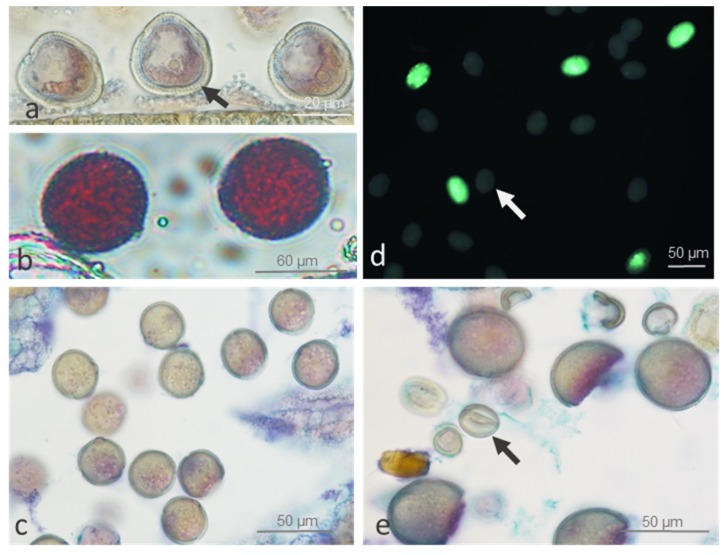
Viable and non-viable microspores and pollen grains of common buckwheat cv. ‘Panda’ and strain PA15 under control (20 °C) and thermal stress (30 °C) conditions. Vacuolated microspores of ‘Panda’ at 20 °C with thick sporodermis (**a**, arrow); viable pollen grains of PA15 at 20 °C stained with Alexander dye (**b**) and at 30 °C stained with Ehrlich’s hematoxylin combined with Alcian blue (**c**); and viable and non-viable (arrows) pollen grains of ‘Panda’ at 30 °C stained with fluorescein diacetate (**d**) and at 20 °C stained with Ehrlich’s hematoxylin combined with Alcian blue (**e**). At least 7900 pollen grains per treatment were analyzed in two replicates.

Pollen viability was generally high (>82%) in cv. ‘Panda’ and strain PA15, independent of temperature, suggesting regular meiosis. Once released from the tetrad (after meiosis), the microspores enlarged, forming vacuoles and thick sporodermis (Figure 1a). Then, they developed into pollen grains. Mature (three-celled) viable pollen exhibited dense cytoplasm (Figure 1b–e) and a positive purple-red or green stain in Alexander and fluorescein diacetate (FDA) tests, respectively (Figure 1b,c). ‘Panda’s’ flowers showed significantly lower pollen viability at 20 °C (82%) than that of strain PA15 (96.3%) (compare Figure 1b vs. Figure 1e). We found no significant influence of thermal stress on pollen viability in either genotype compared with that of the control (compare Figure 1c vs. Figure 1d).

#### 2.1.2. Ovule Development

**Figure 2:**
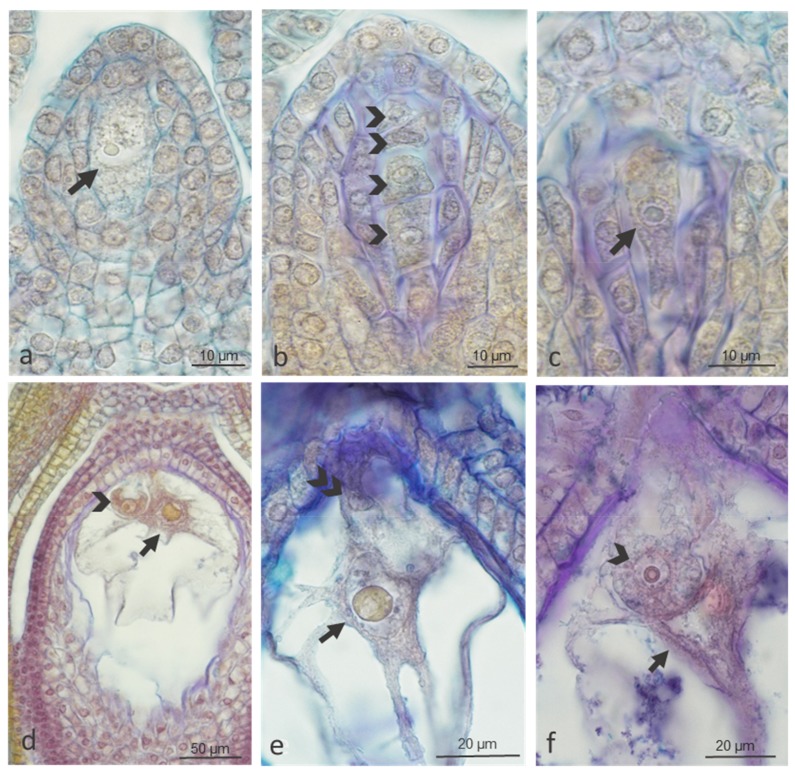
Proper megasporogenesis and female gametophyte development (megagametophytogenesis) at three stages of flower development in ‘Panda’ and PA15 genotypes of common buckwheat under control (20 °C) and thermal stress (30 °C) conditions. Megaspore mother cell in prophase I in ‘Panda’ bud at 20 °C (arrow, **a**), tetrad of megaspores in ‘Panda’ bud at 20 °C (each of four cells marked with arrowhead, **b**), 1-nucleate embryo sac of PA15 at 20 °C (arrow, **c**), central cell (arrows), egg cell (arrowheads), one of the two synergids (double arrowhead) of 7-celled embryo sac in open and wilted flowers (**d**–**f**) of ‘Panda’ at 30 °C (**d**), ‘Panda’ at 20 °C (**e**), and PA15 at 30 °C (**f**). Paraffin sections stained with Ehrlich’s hematoxylin combined with Alcian blue. The number of analyzed flowers per treatment at each stage of development was 15–32 in two replicates.

We observed regular megasporogenesis and different stages of female gametophyte development in buds, open flowers, and wilted flowers of both genotypes (Figure 2). In buds, we noticed clear influence of temperature in cv. ‘Panda’ but not in strain PA15, in which embryological processes were slightly impaired at both 20 °C and 30 °C (83.3% and 79.5%, respectively) (Table 1). In open flowers, the number of properly developed embryo sacs at the control temperature slightly decreased in both genotypes (88.2% in ‘Panda’ and 77.8% in PA15), and a strong impact of high temperature on female gametophyte development was evident in both genotypes. In wilted flowers, this effect was not so clear, probably because flowers with degenerated embryo sacs had been earlier aborted (Table 1).

**Figure 3:**
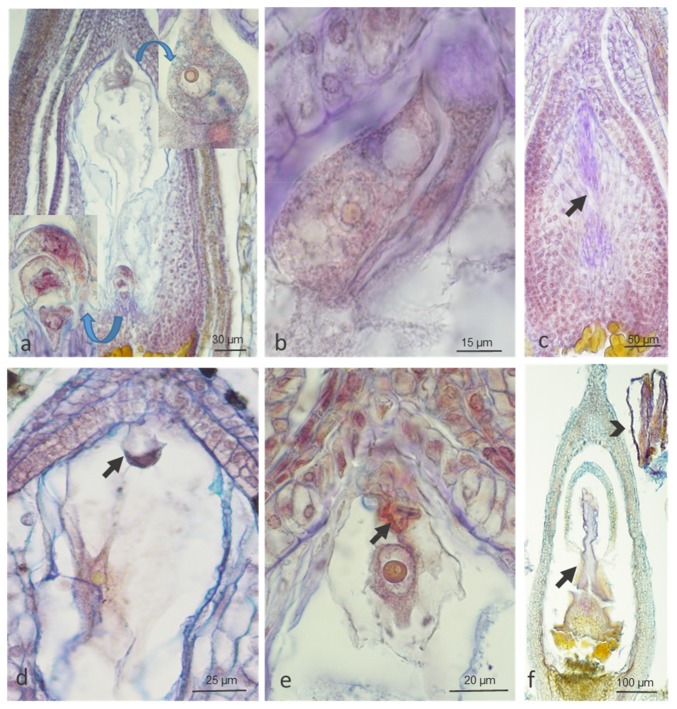
Abnormal female gametophyte development (megagametophytogenesis) at three stages of flower development in ‘Panda’ and PA15 genotypes of common buckwheat under control (20 °C) and thermal stress (30 °C) conditions. Abnormal vacuolization of egg cells of ‘Panda’ (upper insert in (**a**)) and of PA15 open flowers at 30 °C (**b**), at the chalazal pole three antipodal cells visible (lower insert in **a**); degeneration of 2–4-nucleate embryo sac of ‘Panda’ bud at 20 °C (arrow, **c**), of egg cell in PA15 open flowers at 20 °C (arrow, **d**), and of egg apparatus (arrow, **e**), ovule (arrow, **f**), and stamens (arrowhead, **f**) of ‘Panda’ open flowers at 30 °C. Paraffin sections stained with Ehrlich’s hematoxylin combined with Alcian blue. The number of analyzed flowers per treatment at each stage of development was 15–32 in two replicates.

Of the abnormal development patterns of female gametophyte observed under both temperatures, improper position of the nucleus in relation to the vacuole in the egg cell, degeneration of the entire embryo sac, and degeneration of the cells of the embryo sac and of entire ovules were the most frequent (Figure 3).

### 2.2. Hormonal Profile

#### 2.2.1. Total Hormone Content

We determined the hormone content in the flower buds, open flowers, and wilted flowers and in the donor leaves. Table 2 presents the total contents of the studied hormones in the plant organs for strain PA15 and cv. ‘Panda’ at both temperatures. We decided to show the differences between hormone amounts in particular organs independently of growth conditions, as such data are lacking in the literature.

Leaves accumulated the lowest amounts of cytokinins (zeatin and kinetin) as compared with buds and open and wilted flowers. Active forms of gibberellin (GA) and non-active GA_8_ occurred mainly in the open and wilted flowers. Among non-active forms of gibberellins, GA_9_ was the most abundant, and it was detected mainly in the open flowers. This gibberellin also constituted the greatest share in the total pool of gibberellins. Non-active GA_20_ was present in scarce amounts in the flowers but reached its highest level in the leaves. The greatest amount of indole-3-acetic acid (IAA) was found in the wilted flowers, whereas the leaves contained only minute amounts of this hormone. Open flowers accumulated greater amounts of active abscisic acid (ABA-free) than the other organs. In contrast, leaves contained the highest levels of ABA glucosyl ester (ABA-glc). All the developmental stages of flowers showed higher levels of salicylic acid (SA) than leaves, whereas leaves were the richest in jasmonates. The results described above prompted us to conclude that it is difficult to state how or if hormone contents in the donor leaves affect hormone contents in the flowers.

GA_6_ and GA_1_ were the most common in the total pool of active gibberellins in all studied organs, whereas GA_7_ level was the lowest (Table 3). No significant differences in percentage content of particular gibberellins in cv. ‘Panda’ and strain PA15 were observed.

#### 2.2.2. Hormone Content in the Flowers and Leaves

**Figure 4:**
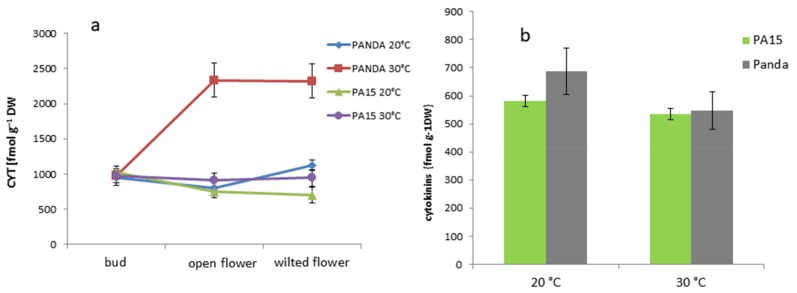
Content of cytokinins (CYT; zeatin and kinetin) [fmol g^−1^ DW] in the flower buds, open flowers, and wilted flowers (**a**) and the leaves (**b**) of the ‘Panda’ and PA15 plants grown at 20 °C (control) and 30 °C (thermal stress). Means (*n* = 3) ± SE.

The content of cytokinins (CYT) in the buds, open flowers, and wilted flowers was similar in the PA15 plants at both temperatures and ‘Panda’ plants grown at 20 °C (Figure 4a). Only ‘Panda’ flowers at 30 °C accumulated considerably greater amounts of CYT, whereas CYT in the buds was still similar to the other objects. Leaves of all the plants showed similar CYT levels at both temperatures (Figure 4b). 

**Figure 5:**
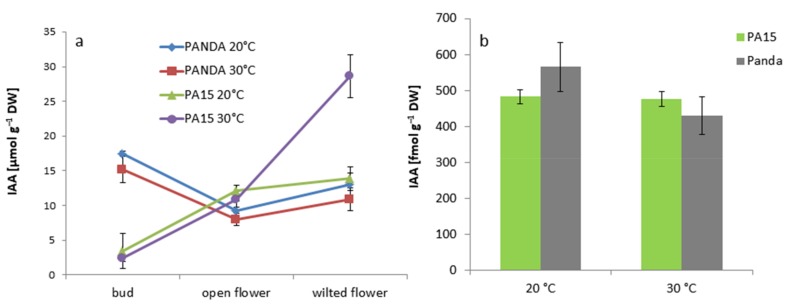
IAA [µmol g^−1^ DW] in the flower buds, open flowers, and wilted flowers (**a**) and the leaves (**b**) of the ‘Panda’ and PA15 plants grown at 20 °C (control) and 30 °C (thermal stress). Means (*n* = 3) ± SE.

The IAA level increased drastically in the wilted flowers of the PA15 plants exposed to thermal stress (Figure 5a), but in the other cases, high temperature did not change the amount of this hormone. The IAA level in flower buds of cv. ‘Panda’ at both temperatures was significantly higher than that in the PA15 plants. We found no effects of temperature on IAA amounts in the leaves of any of the studied plants (Figure 5b).

**Figure 6:**
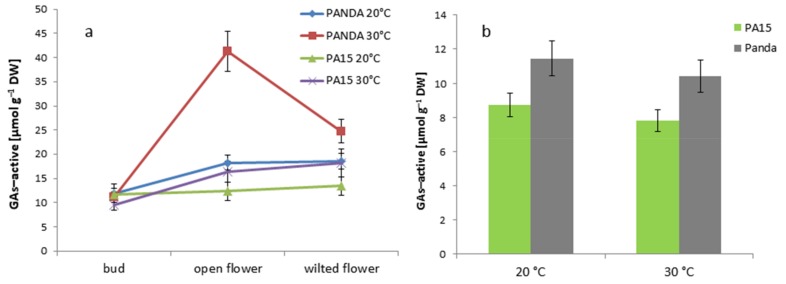
GAs [µmol g^−1^ DW] in the flower buds, open flowers, and wilted flowers (**a**) and the leaves (**b**) of the ‘Panda’ and PA15 plants grown at 20 °C (control) and 30 °C (thermal stress). Means (*n* = 3) ± SE.

The amount of active gibberellins—GA_1_, GA_3_, GA_4_, GA_5_, GA_6_, and GA_7_ (GAs active)—was greater in the well-developed and wilted flowers of PA15 and ‘Panda’ than in the flower buds (Figure 6a). The thermal stress caused a drastic increase in GA levels in the open flowers of ‘Panda’, and a significant decline in the wilted flowers.

The GA level in the leaves of cv. ‘Panda’ was higher at both temperatures than in the PA15 leaves, but thermal stress did not affect the GA amounts in either genotype (Figure 6b). 

**Figure 7:**
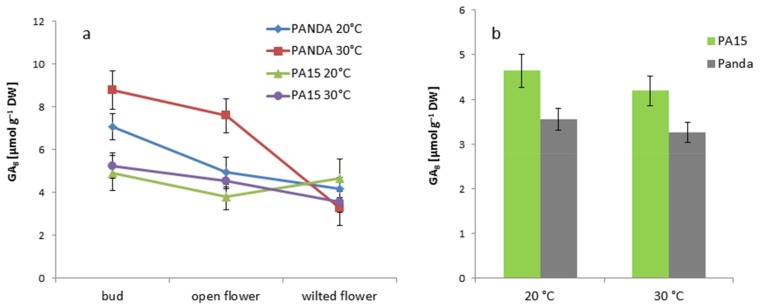
Content of non-active gibberellin GA_8_ [µmol g^−1^ DW] in the flower buds, open flowers, and wilted flowers (**a**) and the leaves (**b**) of the ‘Panda’ and PA15 plants grown at 20 °C (control) and 30 °C (thermal stress). Means (*n* = 3) ± SE.

Generally, greater amounts of non-active GA_8_ were detected in the buds than in the flowers, except for in the PA15 plants grown at 20 °C (Figure 7a). Only in cv. ‘Panda’ did high temperature significantly increase this GA form in the buds and open flowers. The leaves of cv. ‘Panda’ accumulated less GA_8_ than did the PA15 leaves, but we noticed no changes in the hormone levels following the exposure of all the plants to 30 °C (Figure 7b).

**Figure 8:**
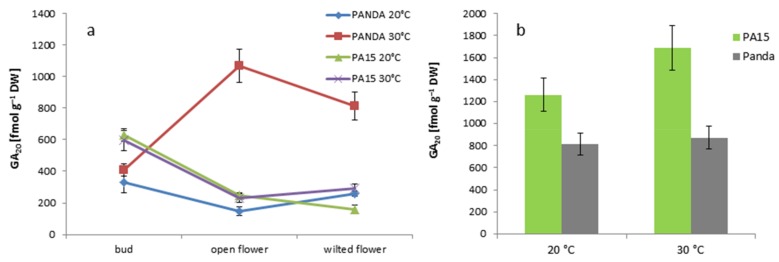
Content of non-active gibberellin GA_20_ [fmol g^−1^ DW] in the flower buds, open flowers, and wilted flowers (**a**) and the leaves (**b**) of the ‘Panda’ and PA15 plants grown at 20 °C (control) and 30 °C (thermal stress). Means (*n* = 3) ± SE.

Another non-active gibberellin, GA_20_, occurred in the flowers in lower amounts than GA_8_ (Figure 8a).

The PA15 plants accumulated more GA_20_ in the buds than in the flowers, and high temperature increased the amount of this hormone only in the wilted flowers. In cv. ‘Panda’, thermal stress triggered more than a five-fold increase in GA_20_ in the well-developed flowers and a two-fold increase in the wilted flowers as compared with the buds. Contrary to cv. ‘Panda’, the leaves of PA15 demonstrated an increase in GA_20_ at 30 °C.

**Figure 9:**
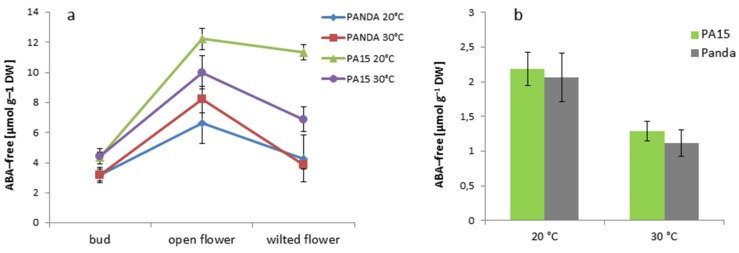
Content of ABA-free [µmol g^−1^ DW] in the flower buds, open flowers, and wilted flowers (**a**) and the leaves (**b**) of the ‘Panda’ and PA15 plants grown at 20 °C (control) and 30 °C (thermal stress). Means (*n* = 3) ± SE.

Changes in the ABA-free content in the buds, open flowers, and wilted flowers followed a similar pattern in both PA15 and cv. ‘Panda’ plants (Figure 9a).

**Figure 10:**
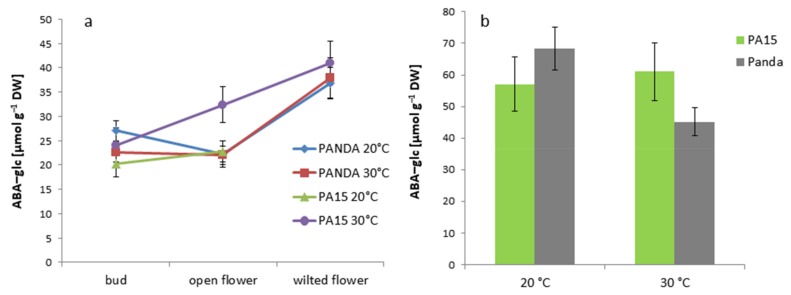
Content of ABA-glc [µmol g^−1^ DW] in the flower buds, open flowers, and wilted flowers (**a**) and the leaves (**b**) of the ‘Panda’ and PA15 plants grown at 20 °C (control) and 30 °C (thermal stress). Means (*n* = 3) ± SE.

The buds of all the plants accumulated the lowest amount of the active form of ABA at both temperatures. Then, the amount increased significantly in the open flowers and decreased in the wilted flowers, except for in the PA15 plants grown at 20 °C. ABA-free was the most abundant in the open and wilted flowers of strain PA15 at 20 °C. Only in the PA15 plants did thermal stress reduce the ABA-free content in the open and wilted flowers. The leaves of all the studied plants grown at 30 °C showed significantly lower amounts of this hormone than those grown at 20 °C (Figure 9b). The amount of ABA-free was similar in the leaves of all the plants regardless of the temperature. Changes in the content of the non-active form of ABA (ABA-glc) in the flowers showed a reverse pattern as compared with that of ABA-free (Figure 10a).

High temperature considerably enhanced the ABA-glc level only in the open flowers of PA15, and the wilted flowers accumulated more ABA-glc than the buds and open flowers. In the leaves of cv. ‘Panda’, thermal stress reduced the ABA-glc level, whereas no temperature effect was visible in the PA15 leaves (Figure 10b). 

**Figure 11:**
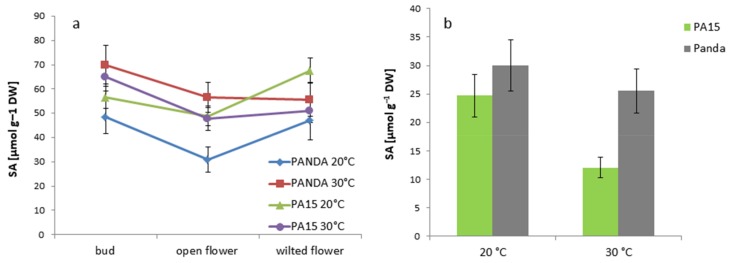
Content of SA [µmol g^−1^ DW] in the flower buds, open flowers, and wilted flowers (**a**) and the leaves (**b**) of the ‘Panda’ and PA15 plants grown at 20 °C (control) and 30 °C (thermal stress). Means (*n* = 3) ± SE.

The salicylic acid profiles in the buds and flowers at both temperatures were similar in the PA15 and ‘Panda’ plants (Figure 11a).

Its lowest amount was detected in the open flowers of the ‘Panda’ plants grown at 20 °C. High temperature increased the SA content mainly in the open flowers of all the studied plants. In the leaves of the PA15 plants, thermal stress reduced the SA content, contrary to that in the leaves of cv. ‘Panda’, where no effect of high temperature was observed (Figure 11b). 

**Figure 12:**
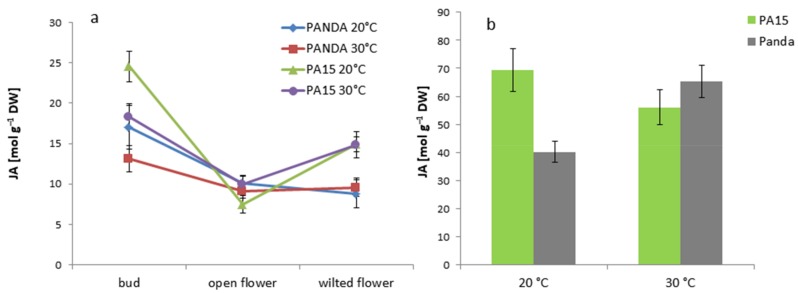
Jasmonic acid (JA) [µmol g^−1^ DW] in the flower buds, open flowers, and wilted flowers (**a**) and the leaves (**b**) of the ‘Panda’ and PA15 plants grown at 20 °C (control) and 30 °C (thermal stress). Means (*n* = 3) ± SE.

The jasmonic acid (JA) level was much higher in the buds of the PA15 and ‘Panda’ plants at both temperatures than those at the other stages of flower development. However, the buds at 30 °C contained less JA than the control buds (Figure 12a).

The lowest JA amount was detected in the open flowers of all the plants at 20 °C and 30 °C. In contrast with cv. ‘Panda’, the amount of JA in the wilted flowers of the PA15 plants grown at both temperatures was greater than that in the open flowers. Additionally, the leaves of PA15 and cv. ‘Panda’ responded differently to high temperature: in the PA15 leaves, the JA amount declined, but in the ‘Panda’ leaves, it was higher than that at 20 °C (Figure 12b). In general, JA was more abundant in the leaves than in the flowers of all the plants. 

**Figure 13:**
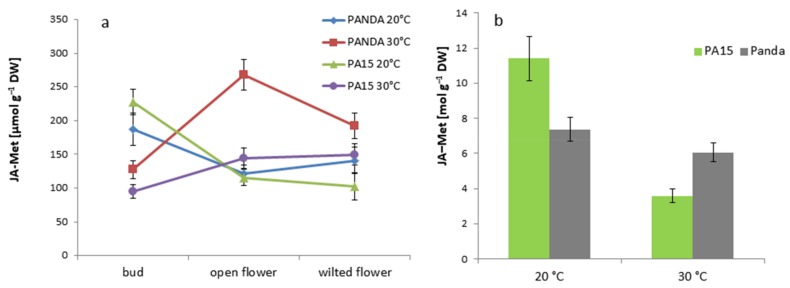
JA [fmol g^−1^ DW] in the flower buds, open flowers, and wilted flowers (**a**) and the leaves [µmol g^−1^ DW] (**b**) of the ‘Panda’ and PA15 plants grown at 20 °C (control) and 30 °C (thermal stress). Means (*n* = 3) ± SE.

The amounts of methyl ester of JA (JA-Met) detected in the flowers (expressed as fmol g^−1^ dry weight (DW)) were much lower than those in the leaves (expressed as µmol g^−1^ DW) (Figure 13). We noticed huge differences in the accumulation of this JA form at different temperatures. In the PA15 flowers of all the developmental stages, no significant temperature effect on JA-Met amount was visible, whereas in the ‘Panda’ flowers, high temperature increased its level as compared with that of the control. The highest amount of JA-Met was detected in the ‘Panda’ open flowers exposed to high temperature. In the leaves of all the studied plants, thermal stress drastically lowered the JA-Met amount.

#### 2.2.3. Quantitative Ratio of the Studied Hormones

Taking into account the role of individual hormones in the generative development of plants at the applied temperatures, we calculated the ratio of active GAs to ABA-free, IAA to ABA-free, and IAA to CYT (Table 4).

Gibberellins were more abundant than ABA-free in all the organs of the PA15 and ‘Panda’ plants at both temperatures. In all the organs, thermal stress significantly increased the amount of active gibberellins in relation to ABA-free, and thus, the GAs/ABA-free ratio increased. The ratio was the highest in the ‘Panda’ open flowers at 30 °C. High temperature significantly boosted the IAA/ABA ratio mainly in buds (six times for PA15 and 10 times for ‘Panda’) and almost doubled it in the leaves. In all the studied plants, CYT levels were much lower than those of IAA. A significant advantage of auxin over cytokinins was observed at 30 °C in the buds of PA15 and cv. ‘Panda’, whereas in the open and wilted flowers, IAA prevailed over CYT at 20 °C. In the buds of all the studied plants at 20 °C, the IAA/CYT ratio was much lower than those in the open and wilted flowers, whereas at 30 °C this proportion was reversed. In the ‘Panda’ flowers, the IAA/CYT ratio at higher temperature decreased to a greater extent than that in the PA15 flowers. High temperature did not affect the IAA/CYT ratio in the leaves.

## 3. Discussion

Our earlier studies showed that the percentage of developmental disturbances occurring in mature female gametophytes resulting from premature degeneration of synergids or the egg apparatus in flowers of plants grown in natural conditions amounts to 40%–55% and depends mainly on the genotype [8]. It seems, therefore, that the key reason for the low yield is the defective development of female gametophytes, taking into account that the anthers of buckwheat produce viable pollen (>90%) [8]. Pollen is formed earlier than the female gametophytes, when plants still have abundant assimilates and air temperature is not high. Therefore, these factors do not affect microsporogenesis and microgametophytogenesis (data not shown). In contrast, the developmental processes in megasoporogenesis and megagametophytogenesis happen later and occur in older flowers and at higher temperatures; therefore, they may be affected by the availability of assimilates and temperature. 

McGregor [17] revealed an approximate flower abortion rate of 45% in *Brassicaceae*. The cause of this phenomenon may be the genetic background or the influence of abiotic factors, such as temperature. For example, abortion causes smaller yields, especially in legumes [18]. Patric [19] suggests that in *Vicia faba* L., minor apical auxin probably regulates the disintegration of generative organs as a result of competition for assimilates rather than through polar and basal transport to the lower parts of the main shoot. Some plant species have the ability to abort low-quality embryos selectively, which raises the average quality of the surviving offspring [20].

According to Moe [21], the process of floral abortion is initiated during the early stages of shoot growth before the differentiation of floral parts is completed. Low temperatures (12–15 °C) at this critical stage of development strongly promote blind shoot formation but do not affect stamen and pistil primordia formation in the apical flower bud. Apart from genetic control, the course of embryogenesis and the abortion of flowers and fruit is also influenced by plant hormones and growth regulators, and in particular the relationship between their concentrations [22]. 

In this experiment, we analyzed the changes in individual hormone content during buckwheat flower development from buds to wilting. Our attempt to explain the disturbances in the embryological development of buckwheat by changes in the hormonal profile of the flowers is an innovative approach and may account for poor seed yielding of buckwheat. The hormone content in individual organs of common buckwheat has not been reported yet.

We found numerous differences in hormone accumulation in common buckwheat organs. Contrary to ABA-free, ABA-glc was present in greater amounts in the leaves than in the flowers. Similarly, as reported by Wang and Irving [22], a higher level of jasmonates accumulated in the donor leaves than in the flowers. According to these authors, the insufficiency of jasmonic acid affects anther or ovule development and results in sterile flower organs. This is probably the reason for embryo sac degeneration in buckwheat plants grown under thermal stress. 

Our results showed that both studied genotypes differ in their response to thermal stress. Cultivar ‘Panda’ seems to be more sensitive to high temperature than strain PA15, as its response to thermal stress was more rapid. Only in the ‘Panda’ buds did high temperature drastically increase the percentage of embryo sac degeneration. In the open flowers of ‘Panda’ at 30 °C, the number of degenerated embryo sacs doubled, and in the open flowers of PA15, the percentage of properly developed embryo sacs was 2.7 times lower than that of the control. These processes were accompanied by an increase in the content of cytokinins, active gibberellins, and JA-Met. Cytokinins control cell division and organ differentiation, for example, while gibberellins stimulate plant elongation and promote flowering. In *Prunus avium*, endogenous GA induced early embryo sac development, which resulted in a low seed set under high temperature [23]. Methyl jasmonate and jasmonic acid are important cellular regulators mediating diverse developmental processes, such as seed germination, flower and fruit development, leaf abscission, and senescence [24]. Interestingly, particularly in the ‘Panda’ ovules at 30 °C, female gametophytes appeared to be extremely luxuriant, but detailed analysis revealed their abnormal vacuolization. Abnormal vacuolization of common buckwheat embryos was observed at 32 °C in Japanese cultivars, displaying only 30% seed set [25,26]. 

Buckwheat flowers and leaves contain active and non-active forms of gibberellins. GA_9_ occurred in the largest quantities of all the determined gibberellins. This gibberellin is a precursor in the GA_1_ biosynthesis pathway [27]. Gibberellins GA_6_, GA_1_, and GA_3_ occurred in larger quantities than other active gibberellins. The profiles of individual active gibberellins in buds, open flowers, and wilted flowers at both temperatures were similar. For this reason, we presented the changes in total sum of active GAs. Halińska and Lewak [28] and Chien et al. [29] reported that a combination of some gibberellins had a greater effect on plant growth and development than individual gibberellin levels. 

ABA is a phytohormone affecting many physiological processes. Its role in flowering promotion is ambiguous. There are data showing negative [30], as well as positive [31], regulation of flowering in *Arabidopsis*. In our study, the pattern of changes in ABA content during flower development was concurrent with earlier reports that ABA plays a signaling role in flower senescence [32,33]. According to Aneja et al. [32] and Panavas et al. [34], the levels of endogenous ABA had increased markedly before any signs of senescence became visible and kept rising during petal senescence in such plant species as cocoa and daylily. Flower senescence in some plant species depends on another signal hormone—ethylene [35]. Zhong and Ciafré [36] described *Iris* as a species whose flowers are ethylene-independent and whose senescence is regulated by ABA. In our opinion, common buckwheat could also belong to this group, because a single flower of buckwheat lives very briefly. In the plants we investigated, ABA-free content was significantly greater in all open flowers than in the flower buds and wilted flowers. Only in the PA15 wilted flowers at 20 °C was the ABA-free level still high, and in the other variants, the wilted flowers demonstrated a considerable decrease in this hormone. In the ‘Panda’ open flowers, high temperature increased ABA-free content, which decreased in the open flowers of PA15. 

The GAs/ABA-free ratio in open flowers of cv. ‘Panda’ demonstrated that at a high ABA level at 30 °C, the content of active gibberellins was even higher. Gibberellins and ABA can act antagonistically [37], and their role is very well known, especially in seed dormancy and germination [38]. We also observed changes in the IAA/ABA and IAA/CYT ratios. The IAA/ABA ratio increased drastically at 30 °C in the ‘Panda’ and PA15 flower buds. This could explain the lack of embryological disturbances under thermal stress but only in the PA15 plants. In the ‘Panda’ buds, only 58% of embryo sacs developed properly as compared with the control. In the buds of all the plants, the IAA/CYT ratio increased six times under thermal stress, but in the open flowers, it was lower at 30 °C than at 20 °C. This result seems to be in accordance with data reported for the effects of high temperature in *Arabidopsis*. These conditions trigger an activation of PHYTOCHROME-INTERATING FACTOR 4 (PIF4). This protein regulates auxin biosynthesis at higher temperatures [30,39]. PIF4 genes stimulate GAs accumulation, increase the transcription level of enzymes synthesizing GAs, and lower the transcription level of GAs inactivating enzymes [40]. Auxins and cytokinins also interact on metabolic levels, and auxins rapidly suppress the cytokinin pool [41]. These considerations could be supported by future experiments with developing flower explants treated with exogenous phytohormones.

## 4. Materials and Methods

### 4.1. Plant Material

The study was carried out in common buckwheat plants of a Polish cultivar ‘Panda’ and strain PA15 in phytotronic conditions. Seeds were supplied by Plant Production Facility in Palikije (Małopolska Plant Breeding Station, Polanowice, Poland).

### 4.2. Experimental Treatments

The experiment was carried out in phytotronic chambers. Plants were cultivated in pots (20 × 20 × 25 cm; 9 plants per pot), containing commercial soil substrate (pH = 5.8) mixed 1:1 with perlite (*v*:*v*). Plants were grown for 3 weeks at the control temperature (20 °C) at a humidity of 50%–60% under 16 h photoperiod and 300 μmol m^−2^ s^−1^ of PPFD (photosynthetic photon flux density). Then, half of the plants (all at the vegetative stage) were transferred into a chamber with a temperature of 30 °C (heat stress) and the same humidity and light conditions. Then, from two-month-old plants, the flowers at three developmental phases (buds, open developed flowers, and wilted flowers) were collected, and their embryological development (embryo sacs) and hormonal profiles were analyzed. Pollen viability was determined in open flowers. Additionally, donor leaves (fully developed young leaves, closest to the flower cluster) were collected for hormone analysis.

### 4.3. Measurements

#### 4.3.1. Embryological Analyses

##### Pollen Viability

Initially, several randomly chosen flowers per treatment were taken for pollen viability screening via a FDA test (fluorescein diacetate). FDA dye was prepared according to Dafni and Firmage [42], as follows: 2 cm^3^ 20% sucrose in H_2_O with several drops of stock solution of FDA (2 mg FDA/1 cm^3^ acetone). Freshly stained pollen was kept in a humid chamber for 30 min at 24 °C and afterwards observed with a Nikon E80i microscope with a UV-2A filter. Viable pollen emits yellow-green fluorescence. No counts were performed for the stained pollen. For the pollen viability count using Alexander’s test, 20 open flowers per treatment were randomly collected and fixed in FAA (10 cm^3^ 96% ethanol, 7 cm^3^ H_2_O, 2 cm^3^ 37% formaldehyde solution, 1 cm^3^ glacial acetic acid) solution. Alexander’s dye is a mixture of malachite green staining the cellulose of pollen walls green and acid fuchsin staining the pollen protoplast red [43]. Viable pollen grains appear purple-red, and non-viable pollen grains stain green. At least 7900 (altogether, viable and non-viable) pollen grains per treatment were counted under a Nikon E80i microscope (Tokyo, Japan) in two replicates.

##### Ovule Development

Paraffin sections of ovules were obtained by fixing flowers at three stages of development (buds, fully developed, and wilted flowers) in FAA solution, dehydrating them in increasing series of ethanol and saturating them with chloroform (in increasing proportion—1:3, 1:1, 3:1, 1:0—with absolute ethanol, each for 2 h at room temperature (RT)) and with paraffin dissolved in chloroform (at 57° for several days, until chloroform evaporated). Flowers prepared this way were embedded in paraffin blocks, sliced into 11–15 μm sections on a rotary microtome (Adamas Instrumenten BV, HM 340E, Leersum, Netherlands), and double stained with Ehrlich’s hematoxylin and Alcian blue [44]. Finally, the slides were mounted in Entellane (Sigma-Aldrich, St Louis, MO, USA) and analyzed under a Nikon E80i microscope. The number of analyzed flowers per treatment at each stage of development was 15–32 in two replicates.

#### 4.3.2. Hormone Content Analysis

Selected phytohormones (auxin, active and non-active forms of gibberellins and abscisic acid, kinetin acid, salicylic acid, and jasmonic acid) were assessed according to the procedure described by Płażek et al. [45]. Freeze-dried and pulverized samples of buds, well-developed and wilted flowers, and leaves were extracted (5 min, 30 Hz, MM400, Retch, Haan, Germany) in 1 cm^3^ of an extraction buffer (methanol/water/formic acid, MeOH/H_2_O/HCOOH, 15/4/1 v/v/v) after the addition of an internal standard solution. Samples were centrifuged (3 min 22,000 × *g*, R32, Hettich, Tuttlingen, Germany), supernatant was collected, and the extraction step was repeated twice. The pooled supernatant was evaporated under N_2_, resuspended in 5% MeOHin 1 M HCOOH, and cleaned up on mixed-mode SPE cartridges (BondElutPlexa PCX, Agilent, Santa Clara, CA, USA), as reported by Dziurka et al. [46]. Phytohormones (auxins, cytokinins, gibberellins, abscisic acid, and jasmonates) were analyzed by ultrahigh performance liquid chromatography (UHPLC) using an Agilent Infinity 1260 device coupled with 6410 Triple Quad LC/MS with an electrospray interface (ESI) ion source (Agilent Technologies, USA). Separation was achieved on an AscentisExpres RP-Amide analytical column (2.7 μm, 2.1 mm × 150 mm; Supelco, Bellefonte, PA, USA) at a linear gradient of H_2_O vs. acetonitril with 0.01% of HCOOH. The stable isotope-labeled internal standard of phytohormones consisted of the following: [^15^N_4_]dihydrozeatin, [^15^N_4_]kinetin, [^2^H_2_]gibberellin A_1_, [^2^H_2_]gibberellin A_4_, [^2^H_2_]gibberellin A_6_, [^2^H_2_]gibberellin A_5_ [^2^H_5_]indole-3-acetic acid, and [^2^H_6_]cis,trans-abscisic acid (OlChemIm, Olomouc, Czech Republic). Targeted profiling of phytohormones was conducted using multiple reaction monitoring (MRM) with a comparison with data obtained for pure and stable isotope-labeled standards of investigated compounds. The following hormone forms were determined: IAA, kinetin (KIN), zeatin (ZEA), active gibberellins (GA_1_, GA_3_, GA_4_, GA_5_, GA_6_, GA_7_), non-active gibberellins (GA_8_, GA_9_, and GA_20_), active abscisic acid (ABA-free) (±)-cis,trans-abscisic acid, non-active (±)-cis,trans-abscisic acid glucosyl ester (ABA-glc), SA, JA, and JA-Met. Detailed descriptions are given in Płażek et al. [45]. The data were presented as fmol (femtomol) or μmol g^−1^ DW. Analyses were performed in three replicates.

#### 4.3.3. Statistical Analyses

Two-way analysis of variance (ANOVA) and Duncan’s multiple range test (at *p* < 0.05) were performed using the statistical package STATISTICA 13.0 (Stat-Soft, Inc., Tulusa, OK, USA). Data were presented as means ± SE (standard error). Non-normal distribution data were analyzed using Chi-squared test (χ^2^, *p* < 0.05). 

## 5. Conclusions

Ovules are much more sensitive to thermal stress than stamens in both genotypes. A drastic reduction in the number of properly developed ovules in open flowers is visible at 30 °C, but this temperature does not affect pollen development and wilted flowers. A considerable increase in ABA in open flowers ready for fertilization may serve as a signal inducing flower senescence observed in the next days. Based on the embryological analyses and hormone profiles in flowers, we conclude that cv. ‘Panda’ is more sensitive to thermal stress than strain PA15, mainly due to a much earlier response to this stress involving the disturbances in embryological processes already in the flower buds.

## Figures and Tables

**Table 1:** Percentage [%] of properly developed embryo sacs of cv. ‘Panda’ and strain PA15 at two temperatures.

Organ	cv. ‘Panda’		PA15	
	20 °C	30 °C	20 °C	30 °C
Buds	100.0	57.7 *	83.3	79.5
Open flowers	88.2	44.4 *	77.8	26.7 *
Wilted flowers	83.3	80.0	81.3	78.9

* statistically significant embryo sac development dependence on temperature (*n* = 10) (Chi-squared test; *p* < 0.05).

**Table 2:** Total amount of individual hormones [μmol g^−1^ dry weight (DW)] in the studied organs of common buckwheat. Mean (*n* = 12) ± SE.

Hormone	Buds	Open Flowers	Wilted Flowers	Leaves
CYT	0.98 ± 0.08 ^b^	1.20 ± 0.11 ^a^	1.27 ± 0.12 ^a^	0.59 ± 0.06 ^c^
IAA	9.63 ± 1.72 ^b^	10.07 ± 1.51 ^b^	16.63 ± 2.50 ^a^	0.49 ± 0.07 ^c^
GAs-active	11.08 ± 0.99 ^c^	22.10 ± 2.32 ^a^	18.74 ± 2.06 ^b^	10.76 ± 1.61 ^c^
GA_8_	2.39 ± 1.15 ^b^	6.45 ± 0.97 ^a^	5.22 ± 0.78 ^a^	1.16 ± 0.17 ^c^
GA_9_	6.50 ± 0.98 ^c^	13.86 ± 1.52 ^a^	10.27 ± 1.02 ^b^	6.73 ± 0.84 ^c^
GA_20_	0.49 ± 0.06 ^a^	0.42 ± 0.06 ^a^	0.31 ± 0.05 ^b^	3.91 ± 0.99 ^ab^
ABA-free	3.71 ± 0.53 ^c^	9.27 ± 1.39 ^a^	6.60 ± 0.99 ^b^	1.66 ± 0.25 ^d^
ABA-glc	23.51 ± 3.90 ^c^	24.97 ± 3.75 ^c^	36.27 ± 5.44 ^b^	57.91 ± 8.69 ^a^
SA	59.60 ± 6.88 ^a^	46.04 ± 6.91 ^a^	55.34 ± 8.30 ^a^	23.06 ± 3.46 ^b^
JA	19.21 ± 2.89 ^b^	9.41 ± 1.41 ^c^	11.30 ± 1.70 ^c^	57.76 ± 8.70 ^a^
JA-Met	0.16 ± 0.01 ^b^	0.16 ± 0.02 ^b^	0.15 ± 0.02 ^b^	7.11 ± 1.07 ^a^

CYT—cytokinins (zeatin and kinetin), IAA—indole-3-acetic acid, GAs—active gibberellins: GA_1_, GA_3_, GA_4_, GA_5_, GA_6_, and GA_7_, non-active gibberellins: GA_8_ and GA_20_, ABA-free—active abscisic acid; ABA-glc—non-active abscisic acid, ABA glucosyl ester; SA—salicylic acid; JA—jasmonic acid; JA-Met—methyl jasmonate. Values represent means (*n* = 12) ± SE. Different superscript letters (a, b, c, …) within rows for each hormone indicate significant differences between means (Duncan’s multiple range test; *p* < 0.05).

**Table 3:** Content of individual active gibberellins in relation to the total pool of active gibberellin forms in the buds, open flowers, wilted flowers, and leaves of the cv. ‘Panda’ and PA15 strain plants grown at 20 °C and 30 °C. Means for the buds and flowers (*n* = 9) and means for the leaves (*n* = 3) ± SE.

Active Gibberellin	Buds and Flowers		Leaves	
	cv. ‘Panda’	Strain PA15	cv. ‘Panda’	Strain PA15
GA_1_	30.6 ± 4.7 ^a^	24.3 ± 3.6 ^a^	16.7 ± 2.0 ^b^	18.7 ± 2.0 ^b^
GA_3_	18.3 ± 2.7 ^a^	17.1 ± 2.6 ^a^	20.7 ± 2.4 ^a^	17.2 ± 2.0 ^a^
GA_4_	5.1 ± 0.5 ^a^	4.5 ± 0.5 ^b^	5.7 ± 0.7 ^a^	4.7 ± 0.5 ^b^
GA_5_	8.6 ± 1.0 ^c^	8.3 ± 1.0 ^c^	11.3 ± 1.3 ^bc^	15.1 ± 1.8 ^a^
GA_6_	36.6 ± 5.3 ^b^	44.4 ± 7.7 ^a^	43.6 ± 5.2 ^a^	42.5 ± 5.1 ^a^
GA_7_	0.9 ± 0.1 ^b^	1.5 ± 0.1 ^b^	2.1 ± 0.2 ^ab^	1.8 ± 0.2 ^a^

Different superscript letters (a, b, c, …)) within rows for each hormone indicate significant differences between means (Duncan’s multiple range test; *p* < 0.05).

**Table 4:** Ratios of GAs* to ABA-free, IAA to ABA-free, and IAA to total cytokinins (CYT) in buds, well-developed flowers, wilted flowers, and leaves of common buckwheat plants grown at 20 °C (control) and 30 °C (thermal stress).

Strain/Cultivar	Temp.	Organ			
		Buds	Open Flowers	Wilted Flowers	Leaves
**GAs/ABA-free**					
PA15	20	2.77 ± 0.33 ^bB^	1.12 ± 1.00 ^cC^	1.19 ± 0.09 ^dC^	4.57 ± 0.51 ^bA^
	30	3.56 ± 0.42 ^aC^	3.08 ± 0.27 ^bC^	4.44 ± 0.49 ^bB^	9.19 ± 1.09 ^aA^
‘Panda’	20	2.15 ± 0.25 ^cB^	1.64 ± 0.15 ^cC^	2.69 ± 0.24 ^cB^	5.86 ± 0.65 ^bA^
	30	3.77 ± 0.45 ^aC^	6.68 ± 0.62 ^aB^	6.52 ± 0.71 ^aB^	10.82 ± 1.18 ^aA^
**IAA/ABA-free**					
PA15	20	0.82 ± 0.09 ^bB^	1.10 ± 0.08 ^bB^	2.52 ± 0.28 ^cA^	0.22 ± 0.02 ^bA^
	30	5.55 ± 0.66 ^aA^	1.56 ± 0.09 ^aC^	3.07 ± 0.39 ^bB^	0.45 ± 0.05 ^aD^
‘Panda’	20	0.59 ± 0.07 ^cC^	1.08 ± 0.08 ^bB^	3.94 ± 0.41 ^aA^	0.28 ± 0.03 ^bD^
	30	5.05 ± 0.49 ^aA^	1.07 ± 0.08 ^bD^	3.03 ± 0.29 ^bC^	0.41 ± 0.05 ^aB^
**IAA/CYT**					
PA15	20	3.29 ± 0.34 ^cB^	17.19 ± 1.72 ^aA^	19.89 ± 1.97 ^bA^	0.87 ± 0.08 ^aC^
	30	18.02 ± 2.1 ^aA^	10.29 ± 1.08 ^cC^	13.94 ± 1.45 ^cB^	0.92 ± 0.09 ^aD^
‘Panda’	20	2.65 ± 2.58 ^dC^	13.91 ± 1.35 ^bB^	24.77 ± 2.67 ^aA^	0.87 ± 0.09 ^aD^
	30	15.75 ± 1.74 ^bA^	4.94 ± 0.52 ^dC^	6.92 ± 0.68 ^dB^	0.92 ± 0.09 ^aD^

Values represent means (*n* = 3) ± SE. Different superscript letters (a, b, c, …) within each column for each parameter and organ and different capital letters within each row indicate significant differences between means of each temperature and organ (Duncan’s multiple range test; *p* < 0.05).* Sum of active gibberellins involved: GA_1_, GA_3_, GA_4_, GA_5_, GA_6_, and GA_7_.

## Abbreviations

ABA	abscisic acid
ABA-free	active form of abscisic acid
ABA-glc	abscisic acid glucosyl ester, non-active abscisic acid
CYT	cytokinins
GA	gibberellin
IAA	indole-3-acetic acid
JA	jasmonic acid
JA-Met	jasmonic acid methyl ester
SA	salicylic acid
ZEA	zeatin
